# Semaglutide treatment for type 2 diabetes in a patient with chronic myeloid leukemia: A case report and review of the literature

**DOI:** 10.1515/med-2025-1184

**Published:** 2025-04-23

**Authors:** Yuchen Zhang, Anxin Li

**Affiliations:** Department of Hematology, The Second Affiliated Hospital of Chongqing Medical University, Chongqing 400010, China; Department of Endocrinology and Metabolism, Chongqing University Central Hospital, Chongqing Emergency Medical Centre, 1 Jiankang Road, Yuzhong District, Chongqing 400014, China

**Keywords:** type 2 diabetes mellitus, obesity, glucagon-like peptide-1 receptor agonists, chronic myelogenous leukemia, semaglutide

## Abstract

**Background:**

Comorbid type 2 diabetes mellitus (T2DM), severe obesity, and chronic myeloid leukemia (CML) present therapeutic challenges, especially given the limited data on glucagon-like peptide-1 (GLP-1) receptor agonists in this setting.

**Methods:**

We describe a 33-year-old female with poorly controlled T2DM (HbA1c 10.7%), severe obesity (BMI 47.05 kg/m^2^), and stable CML on tyrosine kinase inhibitor therapy. She received once-weekly semaglutide (0.5–1.5 mg) for 6 months, alongside insulin glargine and dapagliflozin/metformin. Clinical, biochemical, and molecular parameters were monitored.

**Results:**

After 6 months, her HbA1c declined from 10.7 to 5.5%, fasting plasma glucose from 16.2 to 5.3 mmol/L, and body weight decreased by 18 kg. Lipid parameters improved, and molecular analysis confirmed continued CML remission (undetectable BCR-ABL1). The patient experienced only mild, transient gastrointestinal side effects.

**Conclusion:**

In this complex case, semaglutide proved safe and effective for achieving glycemic control and weight reduction without compromising CML stability. These findings suggest that GLP-1 receptor agonists may be a viable therapeutic option for patients with coexisting T2DM, severe obesity, and stable CML, warranting further investigation in broader populations.

## 1 Introduction

Obesity is defined by the Obesity Medicine Association as a chronic, progressive, recurrent, and treatable multi-factorial neurobehavioral disease, with a body mass index (BMI) of 30 kg/m^2^ or greater. This condition is characterized by adipose tissue dysfunction and abnormal physical forces from excessive fat mass, leading to a spectrum of adverse metabolic, biomechanical, and psychosocial health outcomes [1]. Epidemiological evidence suggests a relentless increase in obesity prevalence, with rates more than doubling in over 70 countries since 1980 [2].

Chronic myeloid leukemia (CML) is primarily driven by the Philadelphia (Ph) chromosome, resulting from a translocation that fuses the BCR and ABL genes, producing the BCR-ABL1 fusion protein with enhanced tyrosine kinase activity [3,4]. The advent of tyrosine kinase inhibitors (TKIs) significantly improved survival rates and enabling patients to maintain chronic-phase disease with a normal life expectancy [3,5,6].

Obesity and type 2 diabetes mellitus (T2DM) significantly increase the risk and worsen the prognosis of various hematological malignancies. Obesity is associated with chronic inflammation and metabolic dysregulation, which can create a pro-inflammatory environment conducive to the development of blood cancers [7,8]. Similarly, T2DM contributes to cancer progression through hyperinsulinemia and hyperglycemia via activating pathways that enhance cancer cell proliferation and resistance to treatment [9,10]. The interplay between these metabolic disorders and cancer makes CML management complicate in patients with metabolic issues [7,11]. Thus, integrating metabolic health strategies into cancer care is crucial for optimizing patient prognosis.

Managing T2DM and severe obesity in patients with CML presents significant challenges due to the interplay between metabolic disorders and TKI therapy. CML patients often exhibit comorbidities such as hypertension, diabetes, and dyslipidemia, which can be exacerbated by TKI treatment [12]. The phenomenon of “diabesity,” where obesity and T2DM coexist, complicates management strategies, as many anti-diabetic medications can induce weight gain, further worsening insulin resistance [13,14]. Effective management necessitates the use of anti-diabetic and anti-obesity therapies that do not compromise leukemia control; glucagon-like peptide-1 receptor agonists (GLP-1 RAs) have shown promise in achieving weight loss while improving glycemic control [15]. Additionally, addressing obesity through lifestyle modifications and pharmacotherapy is crucial, as obesity is linked to poorer leukemia outcomes and increased mortality [16]. Thus, a personalized, multimodal approach is essential for optimizing treatment in this patient population.

GLP-1 RAs, including semaglutide enhance insulin secretion, suppress glucagon release, slow gastric emptying, and promote satiety, contributing to glycemic control and weight reduction. Given that CML treatment with TKIs can exacerbate metabolic dysfunction, leading to increased cardiovascular risk, integrating semaglutide into the management of such patients may improve overall health outcomes and quality of life [17]. Additionally, anti-inflammatory properties of semaglutide may modulate the tumor microenvironment and influence CML progression [18]. However, concerns regarding the potential oncogenic effects of GLP-1 RAs highlight the need for careful evaluation in this patient population [19]. Given these multifaceted considerations, documenting and analyzing semaglutide’s effects in patients with stable CML, T2DM, and severe obesity could provide valuable insights into optimizing metabolic and oncologic care in complex clinical scenarios.

In this case report we described the safety, efficacy, and potential benefits of semaglutide in the management of T2DM and obesity in a patient with CML, without compromising the remission status of CML.

## 2 Case presentation

### 2.1 Background

A 33-year-old female was admitted to our institution presenting with recurrent polydipsia over 2 years and worsening glycemic control for 1 month.

### 2.2 Case presentation

Initially, the patient was diagnosed with low-risk CML 4 years prior, exhibiting a EUTOS long-term survival score of 1.502. Her treatment included Imatinib mesylate, and flumatinib mesylate, complicated by elevated transaminase levels. Notably, the BCR-ABL1 p210 fusion gene, initially positive, was undetectable by quantitative reverse transcription polymerase chain reaction (qRT-PCR) a month prior to admission, indicating molecular remission. The patient’s polydipsia was overlooked; however, it worsened over time, leading to a diagnosis of T2DM 1 year ago. She was treated with insulin glargine (28 U, daily), dapagliflozin (10 mg, daily), and metformin (850 mg, twice daily). Dapagliflozin was selected for its glycemic, weight management, and cardiorenal benefits. Despite this regimen, routine glucose monitoring was not performed. Recently, she reported increased appetite and postprandial glucose levels of 18 mmol/L, along with a weight gain of 10 kg within the last year.

Upon admission, the patient weighed 125 kg, with a BMI of 47.05 kg/m^2^ and an abdominal circumference of 138.2 cm. Laboratory tests showed a fasting glucose of 16.2 mmol/L, urine glucose of 3+, and an HbA1c of 10.7%. Body composition analysis indicated a visceral fat area of 194.8 m^2^, total body fat of 54 kg, and a body fat percentage of 45%. The admitting diagnoses included T2DM, CML in remission, class III obesity, hyperlipidemia, and non-alcoholic fatty liver disease. The patient expressed a strong desire for non-surgical weight management. The clinical characteristics are summarized in Table 1.

**Table 1:** Indicators before semaglutide treatment

	Test value	Reference		Test value	Reference
Fasting plasma glucose (mmol/L)	16.2	3.9–6.1	WBC (G/L)	9.25	3.50–9.50
Hemoglobin A1c (%)	10.7	4.0–6.5	Neutrophil count (G/L)	5.91	1.8–6.3
Fasting C-peptide(ng/mL)	3.26	1.10–4.40	Lymphocyte percentage (%)	33	20–50
Urine glucose	3+	Negative	Basophilic granulocyte (%)	0.4	0.0–1.0
Blood β-hydroxybutyric acid (mmol/L)	0.3	<0.9	Immature granular cells	—	—
Blood chemistry			RBC (T/L)	4.94	3.8–5.1
TC (mmol/L)	6.07	0.00–5.18	Hemoglobin (g/L)	146	115–150
TG (mmol/L)	11.51	0.00–1.70	Platelet count (G/L)	226	125–350
HDL (mmol/L)	1.02	1.29–1.55	BCR-ABL1 (p210)	—	—
LDL (mmol/L)	1.34	0.00–3.37	Obesity		
AST (U/L)	39	7–40	Abdominal circumference (cm)/WHR	138.2/1.01	
ALT (U/L)	35	13–35	BMI (kg/m^2^)	47.05	
GGT (U/L)	102	7–45	Visceral fat area (m^2^)	194.8	0–80
Creatinine (µmol/L)	76	35.0–81.0	Body fat content (kg)	54	11.7–18.45
Blood uric acid (µmol/L)	450	155–357	Body fat percentage (%)	45	21–33

TC, total cholesterol; TG, triglyceride; HDL, high density lipoprotein; LDL, low density lipoprotein; AST, aspartate aminotransferase; ALT, alanine aminotransferase; GGT, γ-glutamyl transferase; RBC, red blood cell; WBC, white blood cell; G/L, 10^9^/L; T/L, 10^12^/L; −, negative; BMI, body mass index; WHR, waist hip ratio.

### 2.3 Intervention

A GLP-1 RA, semaglutide, was initiated at a dose of 0.5 mg once weekly, in addition to the patient’s existing insulin and oral antidiabetic regimens. Fenofibrate was prescribed for hyperlipidemia management.

### 2.4 Outcome

After 1 month of semaglutide treatment, the patient demonstrated marked improvements in body weight, BMI, fat mass, and visceral fat area, along with effective glycemic control (Table 2). No hematological abnormalities were detected (Table 3). Over the next 6 months, the semaglutide dose was gradually increased to 1.5 mg weekly, resulting in further weight reduction, normalization of blood glucose and lipid profiles, and a decrease in HbA1c to 5.5%. The patient tolerated the therapy well, reporting only minor gastrointestinal side effects that resolved without additional intervention. Meanwhile, her CML remained in remission, as indicated by stable BCR-ABL1 (IS) levels.

**Table 2:** Indicators of obesity and T2DM after semaglutide treatment

	Reference	Before semaglutide	1 month	3 months	6 months
Weight (kg)		125	120	118	107
Body mass index (kg/m^2^)		47.05	45.17	43.66	40.27
Abdominal circumference (cm)		138.2	135.1	130.2	128.3
Waist hip ratio		1.01	1	0.97	0.96
Visceral fat area (m^2^)	0–80	194.8	189.8	183.3	178.9
Body fat content (kg)	11.7–18.45	54	50.9	52.6	48.3
Body fat percentage (%)	21–33	45	44.1	45.7	43.2
Fasting plasma glucose (mmol/L)	1.10–4.40	16.2	7.9	6.4	5.3
Glycosylated hemoglobin (%)	4.0–6.5	10.7	8	6.6	5.7
Fasting C-peptide (ng/mL)	3.9–6.1	3.26	Not detected	3.24	Not detected
Total cholesterol (mmol/L)	0.00–5.18	6.07	1.91	2.04	2.05
Low density lipoprotein (mmol/L)	0.00–3.37	1.34	2.35	2.28	1.79

**Table 3:** Indicators of CML

	Reference	4 years ago (at the onset)	3 years ago (MMR)	1 month post-treatment	3 months post-treatment	6 months post-treatment
WBC (G/L)	3.50–9.50	34.17	10.34	9.24	9.02	9.42
Neutrophil count (G/L)	1.8–6.3	30.45	6.18	6.29	5.69	5.92
Neutrophils percentage (%)	40–75	89.1	59.8	63.7	60.9	62.8
Lymphocyte percentage (%)	20–50	7	33.3	29.9	32.8	30.1
Basophilic granulocyte (%)	0.0–1.0	5	0.3	0.3	0.4	0.4
Immature granular cells (%)	0	7	0	0	0	0
RBC (T/L)	3.8–5.1	5.24	4.58	4.8	4.71	4.92
Hemoglobin (g/L)	115–150	164	144	139	139	148
Platelet count (G/L)	125–350	463	249	325	298	343
BCR-ABL1 (p210)	Negative	Positive	Negative	Not detected	Negative	Negative
BCR-ABL1 (IS) %	0	3.578	0	Not detected	0	0

RBC, red blood cell; WBC, white blood cell; G/L, 10^9^/L; T/L, 10^12^/L; MMR, major molecular response.

### 2.5 Conclusion

This case illustrates the potential role of semaglutide in managing complex patients with T2DM, severe obesity, and CML. The results suggest that GLP-1 RAs can be safely and effectively incorporated into therapeutic regimens for such patients, offering substantial metabolic benefits while maintaining oncologic control. Further research is warranted to investigate the broader applicability of this treatment strategy in similar clinical contexts.


**Informed consent:** The patient provided written informed consent for her clinical details to be published in this study.
**Ethical approval:** All procedures performed in this case report were in accordance with the institutional research ethics standards and with Helsinki Declaration.

## 3 Discussion

CML primarily affects white blood cells and is characterized by the excessive proliferation of granulocytes. Remarkably, it is the first cancer found to be associated with a specific genetic abnormality [20,21]. The defining feature of CML is the Ph chromosome, resulting from the t(9;22)(q34;q11.2) translocation, which produces the BCR-ABL1 fusion gene. Approximately 98% of CML patients present with the BCR-ABL1 p210 isoform, while the remaining 2% have p230, p190, or other rare variants [22]. The fusion gene involves a disrupted regulatory element linked to a myristoyl group, leading to continuous tyrosine kinase activation – an essential driver of CML pathogenesis [23].

TKIs such as imatinib, dasatinib, nilotinib, and bosutinib serve as first-line therapies for patients with chronic-phase CML [23]. When CML patients have consistent access to TKIs and respond favorably, their life expectancy can be comparable to that of individuals without CML [20].

Monitoring TKI therapy involves assessing hematologic response, cytogenetic changes, molecular dynamics, and ABL kinase domain mutations [21]. Among these, molecular monitoring – primarily performed through qRT-PCR on peripheral blood samples – is crucial. This technique quantifies BCR-ABL1 transcript levels and is considered both the most widely used and the most sensitive method for determining disease burden in CML, with a detection sensitivity ranging from 0.001 to 0.01% [21].

Both diabetes mellitus and obesity are linked to an increased risk of CML. Meta-analyses indicate that patients with T2DM have a 22–33% higher risk of developing leukemia compared to healthy controls [24,25]. In addition, obesity is associated with a 2–5-fold increased risk of CML compared to individuals with a normal BMI [26–28]. Another meta-analysis of prospective studies found that for every 5 kg/m^2^ increase in BMI, the risk of CML rises by 13% [29]. Among patients with CML, approximately 30% have coexisting obesity and hypertension, 11% have diabetes mellitus, and 18% experience dyslipidemia [30].

The coexistence of obesity or T2DM in patients with leukemia is associated with worse prognoses and higher mortality rates compared to leukemia alone [31–33]. Notably, the relative risk of mortality from leukemia increases by 1.17 for every 5 kg/m^2^ increment in BMI [29]. Patients with CML who also present with abnormal glucose and lipid metabolism, as well as hypertension, face an even greater risk of relapse and mortality than those with CML and obesity alone [34], underscoring the critical impact of metabolic disorders on CML outcomes.

There is growing evidence of therapeutic interactions between CML and DM medications. TKIs such as imatinib and dasatinib have been shown to improve pre-existing diabetes and modestly lower blood glucose levels [35–37]. These effects may be partly attributed to elevated plasma adiponectin levels [38]. Adiponectin, a protein secreted by white adipose tissue, appears to be a molecular link between adiposopathy and leukemia development [39]. It possesses anti-inflammatory properties, enhances insulin sensitivity, prevents atherosclerosis, offers cardioprotection, and regulates apoptosis while inhibiting cell proliferation in the myeloid lineage [39,40]. Collectively, these findings underscore adiponectin’s multifaceted role in both metabolic regulation and cancer pathogenesis. Effective weight management in obese individuals delays the progression from prediabetes to diabetes [41] and provides significant therapeutic benefits for T2DM [42]. Building on these observations, it is postulated that weight control may reduce the incidence of CML and improve the prognosis of patients diagnosed with this condition.

Several pharmacological agents, including GLP-1RAs, dual glucose-dependent insulinotropic polypeptide (GIP) inhibitors, sodium-glucose cotransporter 2 (SGLT2) inhibitors, metformin, and amylin mimetics, have demonstrated efficacy in weight management. In overweight or obese individuals with T2DM, GLP-1RAs such as semaglutide or dual GIP inhibitors like tirzepatide are recommended due to their effectiveness in weight control as well as their glycemic and cardiometabolic benefits [43]. The most common adverse effects associated with GLP-1RAs are nausea and diarrhea; however, these symptoms are typically short-lived, mild to moderate in severity, and resolve spontaneously without requiring additional intervention [44].

In this case report, we present a patient with T2DM, severe obesity (BMI 47.05 kg/m^2^), and CML, who was treated with insulin glargine, dapagliflozin, and metformin. Despite these interventions, the patient’s glycemic control remained suboptimal. She expressed a strong desire to lose weight but declined bariatric surgery, prompting consideration of alternative weight-management strategies such as GLP-1RAs, specifically semaglutide.

However, semaglutide’s prescribing information does not explicitly address its use in patients with leukemia, and its safety profile in individuals with concurrent CML, T2DM, and obesity has not been fully investigated. Existing data regarding semaglutide and cancer risk primarily focus on solid tumors, with limited information on hematological malignancies. One systematic review and meta-analysis of 13,330 patients found no association between semaglutide use and increased cancer risk [45]. Additionally, a multicenter, prospective, observational study (SURE Denmark/Sweden) designed to evaluate the real-world effectiveness and safety of once-weekly semaglutide in clinical practice [43]. In this study, a total of 331 adults with T2DM were recruited. These individuals were initiating or continuing semaglutide treatment as part of routine care. Key inclusion criteria typically included a diagnosis of T2DM and a clinical decision (by the treating physician) to start or maintain semaglutide, independent of the study protocol. Patients were followed to assess changes in glycemic control, body weight, and occurrence of adverse events. During the observation period, one case of B-cell lymphoma was documented among these 331 patients. Investigators assessed this event and deemed it unlikely to be related to semaglutide based on temporal factors, clinical judgment, and the patient’s underlying medical history [43]. Nonetheless, more research focusing on hematological malignancies is warranted.

Managing patients with both T2DM and CML involves balancing effective leukemia treatment with metabolic control. TKIs such as nilotinib, imatinib, and dasatinib are standard therapies for CML but have varying effects on glucose metabolism. Studies indicate that nilotinib is associated with an increased risk of diabetes due to its adverse metabolic effects, including insulin resistance and hyperglycemia [46]. A recent study found that 23.8% of patients on nilotinib developed diabetes within 2 years of treatment [47]. On the other hand, dasatinib has shown potential benefits in T2DM patients by reducing insulin resistance and improving glycemic control, potentially facilitating insulin production and decreasing insulin requirements [36]. Therefore, for diabetic patients requiring CML treatment, dasatinib may be a more favorable TKI, while nilotinib should be used with caution and under strict glucose monitoring. First-line T2DM management with metformin is generally safe alongside TKIs, but insulin and sulfonylureas may require dose adjustments. Given the increased cardiovascular risks associated with both T2DM and CML therapies, routine monitoring of blood glucose, HbA1c, and lipid profiles is essential. A multidisciplinary approach involving oncologists, endocrinologists, and primary care providers is crucial in optimizing treatment strategies while minimizing adverse effects.

Given the absence of evidence suggesting an elevated risk of hematological cancer and in light of the patient’s need for effective weight management, semaglutide appeared to be a viable treatment option. After thorough discussions about potential benefits and risks, semaglutide was initiated alongside continued therapy with dapagliflozin and metformin. The dose of insulin glargine was adjusted in response to the patient’s blood glucose measurements. Clinical indicators – including fasting and postprandial glucose levels, as well as HbA1c – were monitored monthly for the first 3 months of treatment. During this period, all measured glycemic parameters showed significant improvement. The patient’s ongoing regimen included semaglutide (1.0 mg weekly), dapagliflozin (10 mg daily), and metformin (0.85 g three times daily). The inclusion of dapagliflozin aligns with international guidelines for T2DM management, particularly in patients with obesity or cardiovascular comorbidities. Its approval in China, the US, and the EU underscores its role as a standard-of-care agent, not an experimental therapy. Notable reductions in body weight, waist circumference, body fat mass, and visceral fat area were observed. Early in the treatment, the patient experienced mild hiccups, which resolved spontaneously without additional intervention. At the 3-month follow-up, a molecular reassessment of the BCR-ABL gene confirmed the absence of leukemic gene mutations or abnormalities, indicating a stable CML profile under semaglutide therapy. After 6 months of continuous treatment, the patient maintained effective glycemic control and achieved further reductions in body weight, waist circumference, body fat mass, and visceral fat area, with a marked improvement in insulin resistance. Repeated genetic testing consistently showed no evidence of the leukemia gene, suggesting that semaglutide therapy did not adversely impact her CML status.

Historically, up to 15–20% of chronic-phase CML cases progressed to accelerated or blast phase annually prior to the era of TKIs. However, with first- and second-generation TKIs, the risk of progression has been substantially reduced, to about 1–2% per year in patients who maintain deep molecular responses [48,49]. In this case, the patient remained on stable TKI therapy, and her BCR-ABL1 transcripts remained undetectable throughout the semaglutide treatment period. From a mechanistic standpoint, GLP-1RAs do not interfere with BCR-ABL1 signaling and have not been linked to increased transformation risk in patients with CML. Indeed, most documented CML transformations arise from underlying disease evolution or TKI resistance rather than from co-administered metabolic agents [50]. Thus, the introduction of semaglutide for obesity and hyperglycemia management does not appear to elevate the likelihood of CML progression in a patient whose disease is molecularly well-controlled. Nonetheless, close monitoring of CML status via regular qRT-PCR assessments remains essential whenever new therapies are added to a treatment regimen.

Previous observational studies and case reports have examined how CML-directed therapies can intersect with diabetes control. Imatinib and dasatinib, for instance, have been linked to modest improvements in hyperglycemia, potentially through mechanisms involving elevated adiponectin levels and enhanced insulin sensitivity. However, other TKIs (e.g., nilotinib, bosutinib) may exacerbate hyperglycemia or dyslipidemia. In terms of antidiabetic regimens, most published cases describe metformin, insulin, or SGLT2 inhibitors in patients with stable CML. These studies generally report that co-treatment is feasible and can help maintain better glycemic control, sometimes facilitated by TKI-induced improvements in insulin sensitivity. Semaglutide (a GLP-1 RA), specifically, has not been extensively studied in this population, making our report one of the few to detail its safe and effective use. Our findings align with the principle that managing obesity and dysglycemia can be crucial to optimizing outcomes in individuals with hematologic malignancies such as CML. Nevertheless, larger observational cohorts and comparative studies are needed to clarify the long-term effects of combining various novel antidiabetic agents with TKIs on both metabolic control and leukemia progression.

## 4 Conclusion

These findings demonstrate that semaglutide can be successfully incorporated into the treatment regimen for patients with coexisting T2DM, severe obesity, and CML, delivering significant metabolic improvements without compromising leukemia control. This case supports the broader applicability of GLP-1 RAs in managing complex patient profiles, while underscoring the necessity of individualized treatment plans and meticulous monitoring of both oncological and metabolic markers.
